# Persistence of pathogens and bacterial community dynamics in tropical soil after application of raw sewage

**DOI:** 10.1038/s41598-023-40718-0

**Published:** 2023-08-18

**Authors:** Marcus Vinícius Araújo Marques, Bruna Coelho Lopes, Thiago Henrique Ribeiro Silvério, Marcos von Sperling, Thiago de Alencar Neves

**Affiliations:** Department of Sanitary and Environmental Engineering, Federal University of Minas Gerais (Universidade Federal de Minas Gerais), Belo Horizonte, Minas Gerais Brazil

**Keywords:** Microbial ecology, Bioinformatics, Soil microbiology

## Abstract

The objective of this work was to evaluate the persistence of faecal indicators and pathogenic organisms (*Salmonella* spp*., Escherichia coli* and viable helminth eggs) and the structure/diversity of bacterial communities in soil receiving raw sewage (RS) for an extended period of application (3 uninterrupted years). In the experimental design, three treatments were defined: (1) Control soil, characterized by the analysis of a composite sample collected in an area of similar soil, but not a recipient of RS (TSC); (2) Soil receiving conventional mineral fertilization, and furrow irrigation with supply water (TW); and (3) Fertirrigated soil with RS applied by furrows (TF). The results of persistence of pathogenic organisms and indicators in TF indicated a sanitary quality similar to the control soil (TSC), thus potentially bringing low risks of contamination with pathogens present in the soil. The presence of viable helminth eggs was not identified in any treatment studied, because of its low concentration in the raw sewage of the studied system. The TW, TF and TSC treatments had 34.8% of bacterial diversity in common. The bacterial composition of the soil showed a predominance of the *Proteobacteria* phylum in all treatments studied; however, TF was the one with the highest relative abundance of this phylum (44.8%).

## Introduction

Raw sewage (RS) is represented by a complex matrix containing nutrients such as nitrogen, phosphorus and other essential elements for plant growth. However, there are public health concerns about the persistence of pathogenic organisms when sewage is applied to the soil. Also, environmental conditions such as temperature, water content, pH, soil composition and presence of competing organisms affect the survival time and their natural decay in the environment^1–3^.

The presence of water is fundamental in controlling soil temperature, making environmental conditions more favourable for microorganisms. Conversely, extreme conditions of low humidity, acidity, and alkalinity (pH < 6.0 or pH > 8.0) tend not to be favourable for the survival of most bacteria in the soil, with the growth and persistence of enteric bacteria generally being more marked in neutral soils. Given the above, under normal conditions, soil becomes an inhospitable environment for the survival of pathogenic bacteria from RS^1^.

The risk of infections by helminth eggs, with the application of residues and wastewater from different sources in agriculture, can happen since they have a greater persistence in the system than other microorganisms. However, the World Health Organization (WHO) guidelines^4^ suggest that unrestricted irrigation can be done with minimal risk if the wastewater has concentrations of less than one helminth egg L^−1^. Seasonality dictates helminths' survival time and concentration in irrigation wastewater and soil. The dry period tends to present a lower concentration of these microorganisms in the medium due to the climatic conditions involved, even though these microorganisms are more resistant to adverse conditions^5^.

The way of treating and handling wastewater, in addition to the correct choice of agricultural crop, considerably reduces risks to public health^6^. The applied dose of an effluent rich in pathogens dictates the persistence time in the medium^7^. The Brazilian tropical climatic conditions demonstrate that *Escherichia coli* is removed within 13 days after RS application^8^.

Soil microbiology is an essential variable in maintaining the quality of the environment, as there is a microbiota responsible for activities such as soil organic matter (OM) decomposition and nitrogen (N) fixation, which are fundamental in the production process. Furthermore, studies show that the application of sanitary sewage in the soil, as much as it can contribute to the release of pathogenic microorganisms to the environment, also contributes with a series of other microorganisms that have a positive action for the functioning of the soil system^9^.

Using wastewater with lower concentrations of pathogenic microorganisms is a favorable condition from a health point of view. However, when sanitary sewage is treated, even the most simplified treatments will lead to the loss of essential nutrients, mainly N and P. Studies have shown that, in a simplified system for reducing pathogens in wastewater, around 80 and 60% of N and P, respectively, have been removed^10^.

According to the legislation of some countries, studies show the importance of adopting a fallow period (waiting or resting) between the last application of contaminated wastewater and consumption of the food product, depending on how it is prepared^4^ During this fallow period, there is a significant reduction in the risk of contamination with potentially present pathogens.

Regarding the study of bacterial communities, research with the 16S segment rRNA genes offers a unique opportunity for in-depth phylogenetic analysis to highlight the breadth of diversity in the various bacterial phyla found in soil, mainly the RS receptors. Research using this technique has shown that the scope of phylogenetic diversity in the ground is broader than what is implicit in the use of approaches based on in vitro cultivation^11^. Although the soil has a remarkably stable diversity of phylum levels, it constitutes a highly diverse ecosystem in terms of the presence of several levels of order, family, genus and species, with several as yet uncultivated strains of the various bacterial phyla (e.g., *Proteobacteria, Acidobacteria* and *Actinobacteria*)^12^. Studies with phylogenetic sequencing, used to identify the soil bacterial community, have addressed the interaction of its physical, chemical and physicochemical characteristics with changes in the abundance of these microorganisms^13–15^. In this sense, research advances in identifying organisms sensitive to soil interactions with the microbial community, creating the possibility of serving as an indicator of soil quality^12,15^.

This work brings as its main innovation the analysis of the use of raw sewage as fertirrigation of agricultural crops for a long period in a microbiological evaluation. The objective of this work was to study the persistence of pathogenic indicators and organisms (*Salmonella spp, Escherichia coli* and viable helminth eggs) in a soil receiving raw sewage (RS) for a long period of application (3 years), and the structure and diversity of bacterial communities after 2.5 years of RS application.

## Materials and methods

The experiment was conducted in an area at the Sewage Treatment Plant of the Minas Gerais Sanitation Company (*Companhia de Saneamento de Minas Gerais—COPASA ETE—Onça*), that treats municipal wastewater from the cities of Belo Horizonte and Contagem, Brazil, located at the geographic coordinates 19°49′20.6″ South and 43°53′46.6″ West, at an elevation of 852 m.

The region has a humid tropical climate, with most rainfall during the summer. The soil in the area was formed from compacted material, and it was therefore not feasible to obtain a proper classification. However, according to the World Reference Base 2014 (Update 2015)^16^, it has similar characteristics to a Technosol. The initial characterization of the soil is shown in Table 1. All laboratory analyzes were carried out according to the methodology described by EMBRAPA^17^, covering the following parameters: hydrogenionic potential in water (pH); electrical conductivity measured in the soil/water suspension in the proportion of 1:2.5 (EC); total nitrogen (N_total_); phosphorus, potassium, calcium, magnesium and available aluminum (P, K, Ca, Mg and Al_Avail_); effective and potential cation exchange capacity (CEC_Eff_ and CEC_Pot_) potential acidity (H + Al); base saturation (V); aluminum saturation (m); global specific mass of the soil (d); soil water content (U); granulometric analysis (G).

**Table 1:** Initial characterization of the surface layer of soils (0–0.05 m) before the implementation of the experimental treatments.

Parameters		Soil content	Parameters		Soil content
pH		7	Al_Avail_	mmol_c_ dm^−3^	1
EC	µS cm^−1^	51	H + Al		7.5
OM	dag kg^−1^	1.1	CEC_Eff_		102
N_Total_	mg kg^−1^	230	CEC_Pot_		109
P_Avail_	mg dm^−3^	2.6	V	%	93
K_Avail_		54	m		1
Ca_Avail_		1,575	d	g cm^−3^	1.26
Mg_Avail_		253	U	kg kg^−1^	0.187
G	% sand	46			
	% silt	37			
	% clay	17			

The experiment was carried out for three years, with a forage plant (*Pennisetum purpureum*) being planted in the soil, where the following treatments were defined: 1) Control soil, characterized by the analysis of a composite sample collected in an area of similar soil, but not a recipient of RS “Raw Sewage” (TSC); 2) Soil receiving conventional mineral fertilization, and furrow irrigation with supply water (TW) from a public drinking water supply system; and 3) Fertirrigated soil with raw sewage applied by furrows (TF).

All methods comply with all relevant institutional, national, and international guidelines and legislation regarding the plants used in the experiment. It should be noted that the plant material used was obtained from an affiliated institute, however it is a commercial species, freely marketed in the country of the experiment (Brazil), not being an endangered or threatened species.

Fertirrigation with RS was performed weekly by applying a dosage equivalent to 300 kg ha^−1^ yr^−1^ of Na^18^. Table 2 shows the physicochemical characterization of the RS used in the fertigation of the TF treatment. All laboratory analyses were performed according to the methodologies proposed by APHA^19^. The experimental area had 0.1 ha subdivided into the 3 treatments. Irrigation and complementing water application to the plots fertigated with RS were also performed weekly, and evapotranspiration was used to calculate the water demands of the plants^20^. The average annual temperature (T_mean_) was 23 °C, average annual relative humidity (RH_mean_) was 61%, and average yearly accumulated precipitation (Pr) was 1353 mm during the experimental period^21^.

**Table 2:** Average concentrations and standard deviations of physicochemical characteristics of raw sewage applied in fertirrigated experimental plots during the 3-year experimental period.

Parameters		Average concentration*
pH		7.7 (± 0.4)
EC	µS cm^−1^	1523 (± 320)
BOD_5_	mg L^−1^	433 (± 143)
COD		760 (± 74)
N		148.9 (± 33)
P		17.8 (± 6)
K		38.5 (± 11)
Na		71.4 (± 21)
Al		4.2 (± 0.4)
Mn		1.1(± 0.1)
Fe		3.3 (± 0.3)
Zn		0.35 (± 0.05)
Ca		21.3 (± 12)
Mg		6.4 (± 1.3)
Cu		< 0.05

*n = 75; values in parenthesis are the standard deviations.

*pH* hydrogenionic potential, *EC* electrical conductivity, *BOD*_*5*_ Biochemical oxygen demand, *COD* chemical oxygen demand, *N* nitrogen, *P* phosphorus, *K* potassium, *Na* sodium, *Al* aluminum, *Mn* manganese, *Fe* iron, *Zn* zinc, *Ca* calcium, *Mg* magnesium, *Cu* copper.

The indicators chosen for analysis were *Salmonella spp*, *Escherichia coli* (*E. coli*), and viable helminth eggs. The first two are indicators of faecal contamination, and the third is a parasitic indicator of more remarkable environmental persistence. They were also chosen because bacteria and helminths have different removal mechanisms in the wastewater and soil. The *E. coli* analysis was performed by the 1603 U.S EPA method^22^, the *Salmonella spp* analysis was performed by the 1682 U.S. EPA^23^, and the count of viable helminth eggs was quantified by the Meyer method^24^.

Sampling was carried out after the 3rd year of RS application in the three subsequent weeks, immediately after the last fertirrigation, to identify better development conditions for the microorganisms. Soil samples were collected using a sterilized auger. Samples were collected at several points along the experimental area, forming a composite sample for each treatment. The depth analyzed was 0–5 cm, as the most significant microbiological activity occurs in this layer, retaining most of the applied microorganisms, according to Balkhair^25^.

The composition and diversity of the microbial community in the soil were investigated by sequencing amplification of the 16S rRNA gene, using the Illumina MiSeq platform, to identify bacteria. The molecular analysis steps were explained in greater detail in Lopes et al.^15^. Sampling was carried out after 2.5 year of RS application, immediately after the last fertirrigation. Soil samples were collected using a sterilized auger, and were collected at several points along the experimental area, forming a composite sample for each treatment, in a depth of 0–0.05 m.

The bioinformatics part of this methodology concerns filtering the genetic sequences found in the studied soils, being filtered to remove primers, sequences smaller than 150 bp, and ambiguous sequences. Sequences with ≥ 97% similarity were assigned the same operational taxonomic unit (OTU) classifications.

With the count of the OTU classification, descriptive statistical analyzes were applied regarding the relative abundance of bacteria (percentage) in the phylum, class, order, and genus level, and the count of units with relative abundance less than 1% was framed in the “Other” classification. The community richness index (Chao1) and diversity indices (Shannon and Simpson) were calculated in Microbiome Analyst^26^.

Analyzes of macronutrients (N, P, and K) and soil organic matter were carried out after 2.5 years of system operation. Principal component analysis (PCA) was used to analyse the correlation of soil parameters with the relative abundance in the phylum level of the applied treatments, utilizing Pearson's correlation with a significance level of 0.05. The XLSTAT free extension to the Microsoft Excel was used.

## Results and discussion

### Evaluation of the persistence of indicators and pathogens in the soil

Table 3 shows the results of the analysis of the persistence of bacteria that indicate faecal contamination (*Escherichia coli*) and pathogenic bacteria of the genus *Salmonella spp.* on the soil surface layer. It is important to point out that these analyzes were carried out immediately after the 3 years of RS application because it was the time when the soil was exposed for the most prolonged period to the contamination of these microorganisms.

**Table 3:** Results of microbiological analyzes (*E. coli* and *Salmonella* spp.) carried out in the surface layer of the soil (0–5 cm) in the three weeks following the end of the application of raw sewage (RS), after three years of application.

Treatments	Weeks after RS application	*E. coli*	*Salmonella* spp.
		MPN g^−1^ of soil	
TSC	1st	< LD	< LD
	2nd	< LD	0.138
	3rd	< LD	< LD
TW	1st	< LD	0.072
	2nd	< LD	< LD
	3rd	< LD	0.072
TF	1st	31	< LD
	2nd	< LD	0.155
	3rd	< LD	< LD

MPN g^−1^—most probable number per gram of soil; TSC—soil control; TW—soil receiving conventional mineral fertilization; TF—soil receiving raw sewage. Limit of detection (LD) of *E. coli* and *Salmonella spp.* of 10 and 0.065 MPN g^−1^, respectively.

Observing Table 3 in the RS application, which usually contains considerable concentrations of thermotolerant coliforms, represented mainly by *E. coli*, there was no detection of this bacterium in the collected soil samples, being detected in only one sampling of the 1st week. Also, it can be seen that the TFN showed a decrease in the concentration of *E. coli* in the soil in the subsequent weeks, remaining below the detection limit imposed by the method (< 10 MPN g^−1^ of soil).

Regarding *Salmonella spp.* the control soil (TSC) presented a maximum value of 0.138 MPN g^−1^; only the analysis of the sample submitted to TF in the 2nd week delivered values higher than the control soil. The results show similar behaviour with the control soil, indicating that the detection of *Salmonella spp.* of the system can be natural from the place of installation of the experiment.

It is important to emphasize that there are no benchmarks for the concentration of *E. coli* in the soil. However, values established in the Brazilian legislation CONAMA no. 498^27^, of the microbiological quality of sewage sludge for agricultural use can be applied in the evaluation of soil sanitary conditions. In this resolution, the best-classified sludge (class A sludge) must have a value of thermotolerant coliforms lower than 10^3^ MPN g^−1^ of total solids. Therefore, it can be considered that the soil of all treatments and at all evaluation times present a low risk of contamination.

Studies with soil decay rates of *E. coli* with application of raw sewage in soil with and without vegetation to supply the nitrogen demand of 300 kg ha^−1^ year^−1^ of the plants were performed. This study also verified that the concentrations of this microorganism tend to decay to a negligible condition in the medium within two weeks after the application of the wastewater to the soil^8^. Furthermore, the researchers stated that fertirrigation proved to be a good technique for the treatment/final disposal of sewage to inactivate this microorganism, which corroborates the results obtained in this work.

The concentration of *Salmonella spp.* established in the legislation of the United States^28^ for class A sludge must present a concentration lower than 0.75 MPN g^−1^ of TS. According to this reference, all analyses of soil samples had lower values than this, suggesting a low risk of contamination.

A study showed that *Salmonella spp.* tends to be more persistent in the soil than *E. coli*, highlighting the importance of the load of these microorganisms applied to the system^29^. There is a variation in the factors that influence the survival time of *Salmonella* spp. in the soil, with the tendency to become negligible in the system over time^30^. Therefore, with the RS dose applied to the soil in this work, the persistence of *Salmonella* spp. can be considered very low.

The presence of viable helminth eggs was not detected in the soil composite samples collected in its superficial layer (0–5 cm), in the experimental plots submitted to all treatments, after three years of RS application. This result can be explained by the low applied load of these nematodes to the soil, providing their high dilution in this system, considering that there are reports of long periods of persistence of these microorganisms^31^.

Another critical factor for not detecting helminth eggs is the dilution provided by the intrusion of rainwater into the sewerage system. In the literature, concentrations ranging from non-detection to up to 200 helminth eggs per liter of sewage have been reported^32^. In a study that analyzed the use of sewage with average concentrations of 2.6 to 2.8 eggs L^−1^, in the irrigation of vegetables in Ghana, the results indicated an average concentration of 3 eggs g^−1^ of soil, in its superficial layer^5^. A study carried out with sewage sludge applied to the soil of Southern Africa and Senegal, known to be rich in viable helminth eggs, showed that the simple act of harvesting the lettuce 30 days after applying the sludge to the soil reduced the risk of contamination to acceptable levels, as established by the WHO^6^.

### Soil bacterial community structures

The sequencing of the 16S rRNA gene was used to evaluate the microbial composition and diversity of the soil, in which a total of 245,675 high quality sequences were quantified, being 17,039 operational taxonomic units (OTU) obtained in the 3 treatments studied (Table 4).

**Table 4:** Estimation of richness (Chao1) and diversity indices (Shannon and Simpson) of 16S rRNA sequencing, from Illumina sequencing analysis, in the soil, after 2.5 years of experimentation.

Treatments	No. of sequences	No. of normalized sequences	OTU	Shannon	Simpson	Chao1
TSC	71,169	71,169	5,558	7.23	0.998	91.52
TW	95,247	71,169	6,052	7.14	0.997	88.91
TF	79,259	71,169	5,429	7.06	0.997	95.59

*TSC* soil control, *TW* soil receiving conventional mineral fertilization, *TF* soil receiving raw sewage.

**Figure 1:**
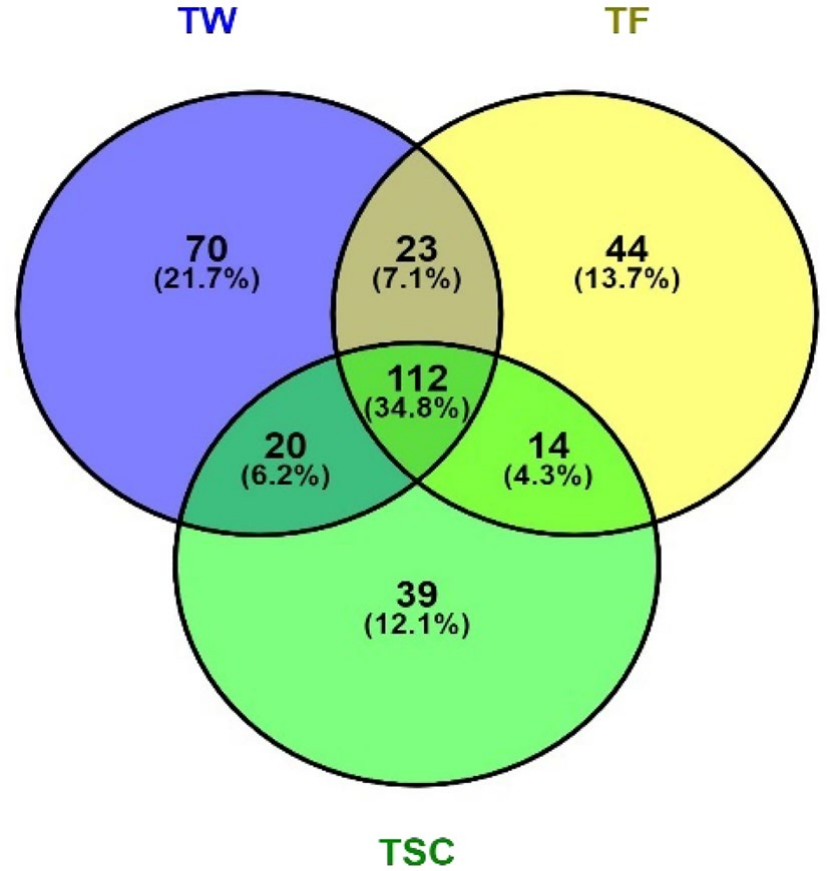
Venn diagram showing the common and unique bacterial taxonomic units (OTU) of the soil receiving raw sewage (TF), soil receiving mineral fertilizer (TW) and the soil control (TSC).

To better understand the composition of the OTU that overlaps within the evaluated treatments, the Venn diagram was applied to display the difference in microbial diversity for each treatment, as shown in Fig. 1.

Comparing, it is noted that the TW presented a higher number of OTU, with 6,052 units identified, being 11.47 and 8.89% greater than the TF and TSC, respectively (Table 4). Observing the Venn diagram, TW presents 21.7% of its bacterial species exclusive to this treatment, while TF and TSC present 13.7 and 12.1% of whole species, respectively (Fig. 1). This exclusivity analysis indicates that mineral fertilization creates conditions for developing a more significant number of species that differ from TF and TSC.

The TW, TF and TSC treatments had 34.8% of bacterial diversity in common; however, TW had 7.1 and 6.2% in common with TF and TSC, respectively. Comparing TF with TSC, they shared 4.3% of bacterial diversity. The study demonstrates the similarity in the bacteriological diversity of soil receiving treated sewage and fresh water, however, there is a more significant number of bacteria associated with nitrification, carbon degradation and faecal indicators in soil receiving treated sewage^9^.

The community richness index (Chao1) points to superiority in the soil of the TF, as a result of the greater microbiological diversity of the RS, and this diversity was reported in other studies^33,34^. Diversity indices (Shannon and Simpson) reflected the negative impacts of TF and TW soil movement. Impacts on microbiological diversity in disturbed soils were presented in the literature in another study^35^.

### Composition of the soil bacterial community

**Figure 2:**
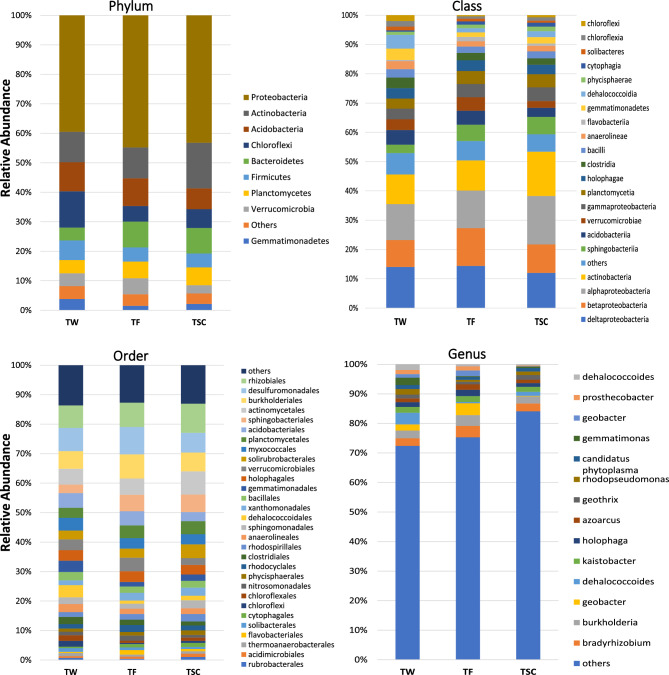
Relative abundance (%) of bacteria at the phylum level, class, order and genus of soil receiving raw sewage (TF), soil receiving conventional fertilization (TW) and soil control (TSC) after 2.5 years of experimentation.

The taxonomic composition of the sample obtained through massive sequencing of the 16S rRNA gene of the bacterial domain is presented in this section. Observing the survey of relative abundance at the phylum level, shown in Fig. 2, it was verified the predominance of the phyla *Proteobacteria*, *Actinobacteria*, *Acidobacteria*, *Chloroflexi* and *Bacteroidetes*, and these represented about 80% of the relative abundance of bacteria in the collected soil samples in the experimental area submitted to all treatments.

There was a predominance of the *Proteobacteria* phylum of 39.5, 44.8 and 43.2%, with relative abundance for TW, TF and TSC, respectively. *Proteobacteria* was reported as the bacterial phylum with the highest relative abundance in other compiled works, with an average similar to those reported in this work (40%), and this phylum presents morphological, physiological, and metabolic diversity that is of great importance for the global cycle of carbon, nitrogen and sulfur in the soil^11^.

In a study carried out with the application of treated sewage, 17 bacterial phyla were observed with *Proteobacteria* (32.1%), followed by *Firmicutes* (26.5%) and *Actinobacteria* (14.3%). These sequences associated with nitrifying bacteria, nitrogen-fixing bacteria, carbon degraders, denitrifying bacteria, potential pathogens, and faecal indicator bacteria were more abundant^9^. These authors concluded that the treated effluent might contain bacteria that can be active in many soil functions and some potential pathogens.

*Actinobacteria* showed considerable relative abundance in the studied soils, being higher in TSC (15%), with an OTU count of 11,000, while in TW and TF, the relative abundance was 10% in both (Fig. 2). This result indicates that soil movement for the crop installation may have been the precursor of this reduction in TW and TF.

In soil, *Actinobacteria* behave like fungi, helping to decompose organic matter and helping to make nutrients available to plants. In addition, some species live symbiotically with the roots that permeate the soil, fixing nitrogen for the plants in exchange for access to some of the saccharides produced by the plants. Also, other genes, such as *Mycobacterium*, are pathogenic and will be confirmed by crossing OTU counts of genes^36,37^.

The *Acidobacteria* phylum showed a different relative abundance and OTU count compared to the *Actinobacteria* phylum, with relative abundance values of 9.9, 9.4 and 7.1% for soil samples collected in TW, TF and TSC, respectively. However, despite this abundance and diversity of *Acidobacteria*, information about their physiology and ecological function remains scarce, mainly due to the low number of cultivable representatives and their slow growth in vitro under standard laboratory conditions^38,39^.

The phylum *Chloroflexi* is deeply branched, containing aerobic and anaerobic thermophilic isolates, filamentous anoxic phototrophs and anaerobic organohalide respirators, which tend to be abundant in the topsoil layer^40^. In this study, the OTU count of the phylum *Chloroflexi* was about twice as high in the TW compared to what was quantified in the soil samples collected in the other treatments. This is due to the increased availability of nutrients that mineral fertilization provides in its successive applications, which does not happen in TF. This factor was verified in pastures that received mineral fertilization from China^13^.

A previous study demonstrates that the *Bacteroidetes* phylum tends to reduce in arable soils, which is responsible for the degradation of complex organic matter^12^. The TW soil had the lowest OTU count (4,120). However, even being a cultivated soil, the TF tended to increase the OTU count (6,913), where the RS created favourable conditions for maintaining and improving the *Bacteroidetes* phylum in the soil due to the incorporation of OM.

Pathogenic organisms, such as *Clostridium*, are part of the *Firmicutes* phylum, but the RS application did not carry these since the OTU count in the TF soil sample was 3,810, while in the TW, it was 6,354. The study demonstrates an increasing trend in OTU counts in arable soils when compared to control soils, relating this increase to different physical–chemical interactions of the soil^12^.

In terms of the dynamics of the communities found in the soil, the *Proteobacteria* phylum occupied the highest abundance in all soils. Studies show that its richness increases with the increase in the availability of OM, and it plays a role in nitrogen fixation in the *Alphaproteobacteria* class, where the *Rhizobiales* order stands out^41–44^. This dynamic is in line with what was found in this study, as TF showed a greater ability to increase with OM (Table 2).

Following the same reasoning, still within the phylum of *Proteobacteria*, the class *Betaproteobacteria*, with emphasis on the *Rhodocyclales* genus, has an important role in the decomposition of inorganic compounds, while the class of Deltaproteobacteria, with emphasis on the *Myxococcales* genus, have the ability to reduce sulfur compounds^41–44^. As previously mentioned, these functions were highlighted in the treatment that received raw sewage.

The *Actinobacteria* phylum, represented by the *Actinobacteria* genus, is recognized for its resistance and high biodegradation capacity, being important in the degradation process of organic and inorganic compounds, with the ability to solubilize phosphorus and potassium in the soil with high agronomic value^44–47^. Therefore, it was expected that this genus would have stood out in terms of abundance in the TF and TW. However, it was more expressive in the TSC, that is, the addition of OM and other nutrients did not cause this genus to stand out. A probable explanation is that this genus is sensitive to the mechanical processes suffered in arable soils and that competition in a favorable environment did not put it in evidence.

A study shows that the presence of heavy metals, such as Zn, Cr, Ni, Cu, Cd and As, can have a pesticidal effect on the development of the *Actinobacteria* and *Proteobacteria* phylum^44^. However, observing Table 2, despite having characterized only Cu and Zn, these are in low concentrations, indicative of the low contribution of heavy metals in the SR used in this study.

The relative abundance at class *level* indicated predominance of *alphaproteobacteria* (12.85%), *deltaproteobacteria* (14.42%), *betaproteobacteria* (12.87%) and *gammaproteobacteria* (4.53%) in the TF treatment soil, being belonging to the phylum *Proteobacteria*. The OTU count of the *betaproteobacteria* class in the TF sample was 10,203, higher than the other treatments. This result can be justified, as *betaproteobacteria* have been reported in significant quantities in various wastewaters^11^.

The *Actinobacteria* class showed a relative abundance of 15% in the TSC, with an OTU of 10,742. This value is numerically higher than the other treatments, and this result can be combined with the non-movement of the soil in this treatment. The *sphingobacteria* class is part of the *bacteroidetes* phylum, with a relative abundance of 2.89% in the TW and an OTU of 1,743 numerically smaller than the other treatments.

Relative abundance at the order level stood out for the order of *rhizobiales*, which presented 7.67, 8.25 and 9.86% of relative abundance for TW, TF and TSC, respectively. The order of *rhizobiales* belonging to the *alphaproteobacteria* class and the phylum *Proteobacteria* thrive in soils with higher amounts of organic carbon^9,48^.

The order of desulfuromonadales showed a relative abundance of 7.86, 9.28 and 6.68% for TW, TF and TSC treatments, respectively. The order desulfuromonadales belongs to the class of deltaproteobacteria and the phylum Proteobacteria, responsible for playing essential roles in the degradation of organic matter and being involved in syntrophic associations, especially with methanogens and phototrophic green sulfur bacteria^49^.

Observing the OTU count at the order level, the burkholderiales had their highest count for the TF (6,507), with a relative abundance of 8.2%. Bacteria of the burkholderiales order can serve as an indicator for effectively removing hydrocarbons at low oxygen concentrations^50^.

The genera of *bradyrhizobium*, *burkholderia* and *geobacter* showed relative abundance in the TF of 3.84, 3.66 and 4.02%, respectively. The genus of *bradyrhizobium* belongs to the proteobacteria phylum, which is composed of bacteria capable of generating nodules on plant roots, which increases nitrogen fixation in the system^51^.

The genus *burkholderia* belongs to the proteobacteria phylum, which is pointed out in studies, as it includes some pathogenic species in this genus. However, many strains are not pathogenic and have desirable characteristics, such as beneficial associations with plants and degradation of pollutants^52^. The genus of *geobacter* also belongs to the proteobacteria phylum and presented its lowest relative abundance in the TSC (0.16%). Nevertheless, the genus *geobacter* has been associated with satisfactory results in bioremediation processes linked to the reduction of sulfur compounds^53^.

**Figure 3:**
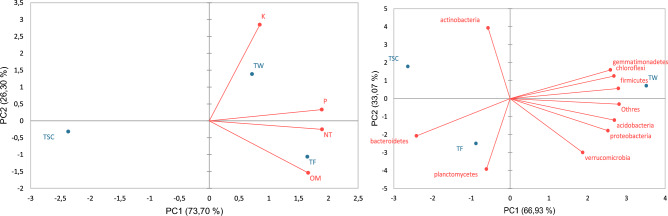
Two-dimensional principal component analysis (PCA) ordination diagram based on macronutrient and organic matter content in soil (left), and the relative abundance of soil bacteria at phylum level (right).

Figure 3 shows the correlation between soil attributes and the relative abundance of soil bacteria as a function of the treatments. In Fig. 3 (left) the parameters that correspond to the X-axis (PC1) were TN, P and OM, while the Y-axis (PC2) was K. Therefore, it is noted that there is a direct positive correlation of the OM, TN and P from the soil for the treatment that received RS (TF), which was expected due to the expressive amount of these elements in the RS, as can be seen in Table 2. The TW also showed a positive correlation with the OM, TN and P, but this not more evident than for TF. The TW showed a positive trend in the amounts of K, largely due to the rapid availability of nutrients via chemical fertilization.

In Fig. 3 (right) the phyla corresponding to the X-axis (PC1) were *Proteobacteria*, *Acidobacteria*, *Chloroflexi*, *Bacteroidetes*, *Firmicutes* and *Gemmatimonadetes*, while to the Y-axis (PC2) were *Actinobacteria*, *Planctomycetes* and *Verrucomicrobia*. Therefore, it is noted that the TW was the one that presented the highest correlation in terms of relative abundance, that is, this correlation is not linked to the addition of OM and TN, which was noted in the TF, and most likely the availability of nutrients from of chemical fertilization.

The TF showed a positive correlation with the *Bacteroides*, which, as previously mentioned, are bacteria responsible for the degradation of complex OM, indicating this correlation of the RS in increasing the OM of the TF. This increase is also due to the presence of this phylum in the human intestinal tract^54^, so RS must have increased its load in the soil.

The TSC showed a positive correlation with the *Actinobacteria*, so if the natural characteristics of the soil are to be observed (Table 1) these do not present expressive favorable conditions for the microbiological development in terms of OM and availability of nutrients. However, the actinobacteria are recognized for their resistance in stressful environments from the microbiological point of view, due to their ability to produce metabolites^47^.

## Conclusion

The persistence of pathogenic indicators in the soil showed a sanitary quality similar to that of the control soil, therefore suggesting low risks of contamination with pathogens present in the soil. The study conducted in the three weeks after RS application in the soil showed no persistence of the indicators over time, as the results of the analyses were below the detection limit or close to the limit. Viable helminth eggs were not detected in the studied soil in any treatment because of the low concentration of helminth eggs in the wastewater and the studied effluent (RS).

The soil receiving conventional fertilization (TW), soil receiving raw sewage (TF) and soil control (TSC) treatments had 34.8% of bacterial diversity are shared. However, TW had 7.1 and 6.2% in common with TF and TSC, respectively. Comparing TF with TSC, they had 4.3% of bacterial diversity in common. The community richness index (Chao1) pointed to superiority in the soil of the TF, as a result of the greater microbiological diversity of the RS. Conversely, diversity indices (Shannon and Simpson) reflected the negative impacts of TF and TW soil movement.

There was a predominance of the *Proteobacteria* phylum of 39.5, 44.8 and 43.2%, with relative abundance for TW, TF and TSC, respectively. The relative abundance at class *level* were *alphaproteobacteria* (12.85%), *deltaproteobacteria* (14.42%), *betaproteobacteria* (12.87%) and *gammaproteobacteria* (4.53%) were predominant in the TF treatment soil, being belonging to the phylum *Proteobacteria*. Relative abundance at order level stood out for the order of *rhizobiales*, which presented 7.67, 8.25 and 9.86% of relative abundance for TW, TF and TSC, respectively.

This work elucidated which are the biodiversity development indices of a soil receiving raw sewage, making it possible to identify which are the phyla, class, order and genus that are present in greater abundance in the soils submitted to the treatments established here. Therefore, you can notice a gain in these parameters with the application of raw sewage, and this practice can be used to recover the microbiology of the soil in degraded areas (Supplementary Information 1, 2, 3, 4).

### Supplementary Information

Supplementary Information 1.

Supplementary Information 2.

Supplementary Information 3.

Supplementary Information 4.

## Data Availability

All data generated or analyzed during this study are included in this published article such as figures, tables, graphs, and supplementary material.
